# Prevalence, Awareness, and Management of Carpal Tunnel Syndrome Among Diabetic Patients

**DOI:** 10.7759/cureus.53683

**Published:** 2024-02-06

**Authors:** Abdullah I Abuharb, Alwaleed I Almughira, Hatan K Alghamdi, Majdi Hashem, Ibrahim Bin Ahmed, Abdulmalik Aloriney

**Affiliations:** 1 College of Medicine, Imam Mohammad Ibn Saud Islamic University, Riyadh, SAU; 2 Surgery, Imam Mohammad Ibn Saud Islamic University, Riyadh, SAU; 3 Family Medicine, Imam Mohammad Ibn Saud Islamic University, Riyadh, SAU; 4 Family Medicine/Diabetes, Imam Mohammad Ibn Saud Islamic University, Riyadh, SAU

**Keywords:** awareness, management, prevalence, diabetes, carpal tunnel syndrome

## Abstract

Background: Carpal tunnel syndrome (CTS) is a common condition that can significantly affect the quality of life for individuals, particularly those with diabetes. This study aims to examine the severity of CTS symptoms among diabetic patients and explore the associations between demographic factors, diabetic characteristics, knowledge, and management approaches.

Methodology: A cross-sectional study was conducted among diabetic patients, and data were collected using standardized questionnaires. The incidence and severity of CTS were assessed using the Boston Carpal Tunnel Questionnaire (BCTQ). Demographic information, diabetic characteristics, knowledge of CTS, and management approaches were also recorded. Descriptive statistics and inferential analysis were performed to analyze the data.

Results: The study included 303 participants. The majority of the participants were aged 50 or older (44.9%) (N=136), followed by those aged 39-49 (24.4%) (N=74). In terms of gender, there were more female participants (61.4%) (N=186) than male participants (38.6%) (N=117). Older age groups exhibited higher mean BCTQ scores, suggesting increased severity. Females had significantly higher severity scores compared to males (mean BCTQ score of 17.52 vs 15.56). Regarding diabetic characteristics, complications in the eye and pain/numbness in the legs or feet were significantly associated with higher severity scores of CTS (P=0.0001). The study revealed a knowledge gap among diabetic patients about CTS, with inadequate knowledge about its causes, symptoms, consequences, and treatment options among 68.6% of the patients. The use of medical interventions such as splints, injections, and surgery was associated with higher severity scores.

Conclusion: This study highlights the incidence and severity of CTS symptoms among diabetic patients and its associations with demographic factors, diabetic characteristics, knowledge, and management approaches. Older age, female gender, widowed, diabetic complications in the eye, and pain/numbness in the legs or feet were found to be related to increased severity of CTS. Additionally, inadequate knowledge about CTS was observed among diabetic patients.

## Introduction

The carpal tunnel is formed by the carpal bones and the transverse ligament. The tendons of the flexor digitorum superficialis, flexor digitorum profundus, and flexor pollicis longus, as well as the median nerve, flow through the tunnel [1]. Carpal tunnel syndrome (CTS) is caused by excessive wrist movement and entrapment of the median nerve within the canal. Nocturnal paresthesia and discomfort are caused by nerve compression. A physical examination will reveal grip weakness, a positive Tinel's sign, a positive Phalen's test, and, if severe, thenar atrophy. Night splinting, over-the-counter anti-inflammatory medicines, corticosteroid injections, and, as a last resort, surgery are all options for treatment [2,3]. There is not a single cause of CTS; nevertheless, several variables contribute to the disease's development. Frequent and repetitive hand motions, such as typing or using a keyboard, are considered the main cause of CTS. Such people may have arthropathies such as arthritis, osteoarthritis, or rheumatoid arthritis. CTS can be caused by hormonal and metabolic disorders such as pregnancy, menopause, thyroid abnormalities, and diabetes mellitus (DM) [4]. Obtaining an accurate history of the patient, performing a physical examination that includes sensory and motor evaluation, performing a nerve conduction study, and electromyography (EMG) in accordance with American Academy of Neurology/American Association of Neuromuscular and Electrodiagnostic Medicine/American Academy of Physical Medicine and Rehabilitation guidelines are all part of diagnosing carpal tunnel syndrome [5]. CTS can be treated using both surgical and non-surgical methods. While surgical decompression is considered the final treatment for CTS, non-surgical therapies such as physical therapy, bracing, steroid injections, and alternative medicine can slow the development of CTS [6]. Keeping all of this in mind, CTS is the most frequent entrapment neuropathy, affecting 3-6% of individuals globally [7]. CTS is the most prevalent compression neuropathy in the general population and is common in persons with type 1 and type 2 diabetes. The reason(s) why a diabetic's peripheral nerve trunk is more vulnerable to nerve compression is unknown, although biochemical and structural changes in the peripheral nerve are most likely involved. Individuals with neuropathy are more prone to acquire peripheral nerve compression illnesses, such as diabetic neuropathy [8]. CTS is more common among diabetics, especially those with concomitant diabetic polyneuropathy (DPN) and/or longer DM duration. It is unclear if it is more frequent in type 1 or type 2 diabetes. Although the precise origin is uncertain, it appears to be hyperglycemia-induced median nerve edema, increased susceptibility to external stress, nerve myelin ischemia, and axonal degeneration. Increased vascular endothelial growth factor (VEGF) and advanced glycation endproducts (AGEs) appear to have a role as well. Median nerve conduction testing, which is more sensitive than clinical examination, remains the cornerstone of CTS diagnosis in diabetes [9]. The association between CTS and DM is not well understood. Therefore, this research aims to fill this gap by studying the prevalence, awareness, and management of CTS symptoms among diabetic patients.

## Materials and methods

Study design

This cross-sectional retrospective study aimed to assess the awareness of CTS symptoms among diabetic patients aged 18 years old and above in Riyadh, Saudi Arabia.

Study sample

The target population for this study was the diabetic general population in Riyadh. The estimated sample size was determined to be between 250 and 350 participants. Participants were included if they met the following criteria: (1) aged 18 or above, (2) diagnosed with diabetes (type 1 or type 2), and (3) residing in Riyadh. Participants were recruited through online surveys posted on various platforms and family medicine clinics in Imam Medical Centre.

Data collection

Data collection was conducted using a self-reported questionnaire consisting of four parts. Part one of the questionnaire collected demographic information, including age, gender, marital status, educational level, and nationality. Part two focused on diabetes-related factors, such as the type of diabetes, duration of diabetes, hemoglobin A1C levels to assess diabetes control (where hbA1c less than 7% is considered controlled [10]), type of medication, presence of kidney, eye, or neurological complications, and vitamin usage.

Part three of the questionnaire assessed participants' knowledge of CTS. It began with a question regarding whether participants had heard of CTS and the source of their knowledge. Subsequently, nine questions were included to evaluate participants' knowledge of CTS, with response options of "yes" (indicating a correct answer), "no" (indicating a wrong answer), or "I do not know" (also indicating a wrong answer). Each correct answer was assigned one point, resulting in a knowledge score ranging from 0 to 9. Participants scoring 5 or above were classified as having adequate knowledge.

Part four of the questionnaire aimed to assess the incidence, prevalence, and severity of CTS symptoms among the participants. The Boston Carpal Tunnel Syndrome Questionnaire (BCTS) was utilized, consisting of 10 questions. Each question was scored on a scale from 1 (indicating low incidence) to 5 (indicating high incidence), resulting in a total score ranging from 10 to 50. A higher score indicated a higher incidence and severity of CTS symptoms.

Data analysis

Microsoft Excel was used for data entry while the Statistical Package for the Social Sciences (IBM SPSS Statistics for Windows, IBM Corp., Version 26.0, Armonk, NY) was used for data analysis. Descriptive statistics were used to summarize the demographic characteristics of the participants. The knowledge scores and BCTS scores were analyzed to assess the awareness and prevalence of CTS symptoms among the diabetic population. Chi-square tests or t-tests were conducted to identify any associations between demographic factors and CTS awareness.

Ethical considerations

This study obtained ethical approval from the institutional review board of Al-Imam Mohammad Ibn Saud Islamic University (IMSIU) project number 432/2023. Participants were assured of the confidentiality and anonymity of their responses. Informed consent was obtained from all participants before their inclusion in the study.

## Results

In the current study, we were able to collect data from 303 diabetic patients. The majority of the participants were aged 50 or older (44.9%) (N=136), followed by those aged 39-49 (24.4%) (N=74). In terms of gender, there were more female participants (61.4%) (N=186) than male participants (38.6%) (N=117). The majority of participants were Saudi nationals (93.7%) (N=284). Regarding education level, the largest group had a bachelor's degree (37.6%) (N=114), while the smallest group consisted of individuals with a doctorate degree (6.3%) (N=19). In terms of marital status, the majority were married (67.0%) (N=203), followed by single participants (23.1%) (N=70) (Table 1).

**Table 1:** Demographic factors of the participants (N=303)

Demographic factors of the participants (N=303)		N	%
Age	18-28	61	20.1%
	29-38	32	10.6%
	39-49	74	24.4%
	50 or older	136	44.9%
Gender	Male	117	38.6%
	Female	186	61.4%
Nationality	Saudi	284	93.7%
	Non-Saudi	19	6.3%
Level of education	Non-learner	32	10.6%
	High school	72	23.8%
	University student	38	12.5%
	Bachelor’s degree	114	37.6%
	Master’s degree	28	9.2%
	Doctor degree	19	6.3%
Marital status	Single	70	23.1%
	Married	203	67.0%
	Divorced	13	4.3%
	Widow	17	5.6%

Table 2 presents information about the diabetic characteristics of the patients. The majority of participants had type 2 diabetes (60.1%) (N=182) and had been diagnosed for more than 20 years (16.8%) (N=51). Diabetic control was reported as uncontrolled for the majority of participants (63.4%) (N=192). The most common type of drug used for diabetes management was oral medications (63.4%) (N=192). Complications in kidney function were reported by 8.6% (N=26) of participants, complications in the eye by 30.4% (N=92), and complications like pain and numbness in the legs or feet by 31.7% (N=96). The frequency of vitamin supplementation varied, with 49.5% reporting occasional use. The majority of participants did not use any medical intervention for their wrist symptoms (89.1%) (N=270). Among patients depending on insulin, insulin aspart (NovoRapid®) was the most commonly used type of insulin reported by 58.2% (N=64) of patients, followed by insulin glargine (Lantus®) (33.6%) (N=37), and insulin glargine U-300 conc (Toujeo®) (20.9%) (N=23).

**Table 2:** Diabetic characteristics of the patients

Diabetic characteristics of the patients		N	%
What type of diabetes do you have?	Type 1	121	39.9%
	Type 2	182	60.1%
What is the duration of the diabetes?	Less than one year	57	18.8%
	1-5 years	56	18.5%
	6-10 years	55	18.2%
	11-15 years	50	16.5%
	16-20 years	34	11.2%
	More than 20 years	51	16.8%
Diabetic control	Uncontrolled	192	63.4%
	Controlled	111	36.6%
What type of drug do you take for your diabetes?	Insulin injections	111	36.6%
	Oral medications	192	63.4%
Were you diagnosed with any complications in your kidney function?	No	277	91.4%
	Yes	26	8.6%
Were you diagnosed with any complications in your eye?	No	211	69.6%
	Yes	92	30.4%
Were you diagnosed with any complications like pain and numbness in your legs or feet?	No	207	68.3%
	Yes	96	31.7%
How often do you take vitamin supplementations like vitamin B?	Never	74	24.4%
	Occasionally	150	49.5%
	Routinely	79	26.1%
Did you use any medical intervention to your wrist symptoms?	I did not use anything	270	89.1%
	Splint	17	5.6%
	Injections around the wrist	10	3.3%
	Carpel tunnel release surgery	6	2.0%
Insulin type (N=111), multi-answer question	Insulin aspart/insulin aspart protamine (Novomix^®^)	8	7.3%
	Insulin aspart (Novorapid^®^)	64	58.2%
	Insulin glulisine (Apidra^®^)	9	8.2%
	Insulin lispro/Insulin lispro protamine (Humalog 25/75^®^)	3	2.7%
	Insulin glargine (Basalgar^®^)	4	3.6%
	Insulin glargine (Lantus^®^)	37	33.6%
	Insulin degludec (Tresiba^®^)	17	15.5%
	Insulin degludec/insulin aspart (Ryzodeg^®^)	4	3.6%
	Insulin glargine U-300 conc (Toujeo^®^)	23	20.9%

**Figure 1:**
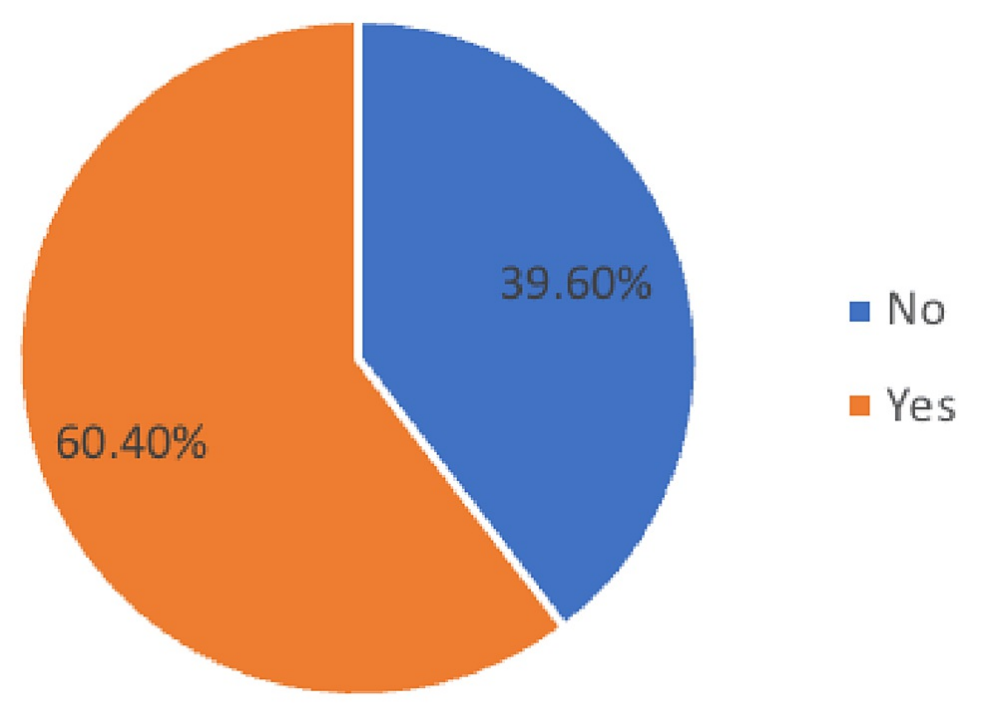
Have you ever heard of carpel tunnel syndrome?

**Figure 2:**
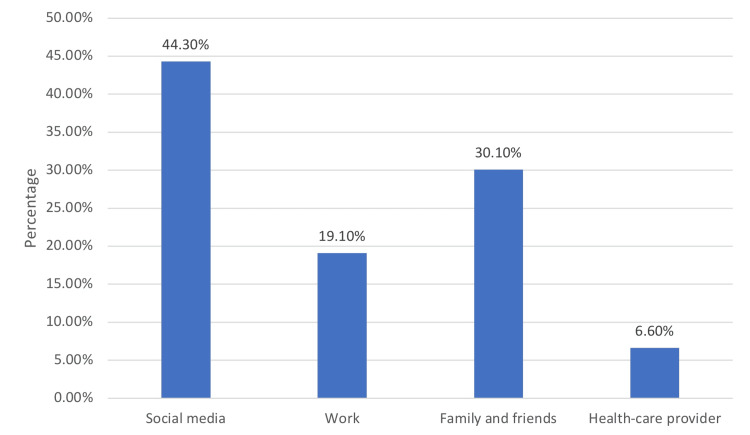
Source of knowledge

In Figure 1, 60.4% (N=183) of participants reported having heard of CTS (Figure 1). Among those who were aware of it, the main source of knowledge was social media (44.3%) (Figure 2).

Participants' knowledge of CTS varied across different aspects. Only 32.7% (N=99) correctly identified CTS as a syndrome caused by median nerve compression, and 37.3% (N=113) knew the common symptoms of pain and tingling in the fingers. Moreover, 27.1% (N=82) recognized the consequences of untreated CTS, and 30.0% (N=91) knew that it could affect both hands. Participants had limited awareness of preventive measures (28.7% (N=87) knew about avoiding repetitive movements as a preventive measure) and treatment options (22.8% (N=69) knew surgical intervention as the main treatment). Overall, the participants' knowledge of CTS was inadequate for the majority (68.6%) and adequate for 31.4% of them (Table 3).

**Table 3:** Knowledge of the participants toward CTS (Yes is the correct answer for all questions) CTS: Carpal tunnel syndrome

Knowledge of the participants toward CTS (Yes is the correct answer for all questions)	No		Yes		I do not know	
	N	%	N	%	N	%
CTS is a syndrome caused by median nerve compression	16	5.3%	99	32.7%	188	62.0%
‏Common symptoms of CTS are pain, tingling of index finger, middle finger and thumb	16	5.3%	113	37.3%	174	57.4%
Stiffness and weakness of the sixth muscle can happen if the condition remains untreated	18	5.9%	82	27.1%	203	67.0%
CTS can affect both hands	22	7.3%	91	30.0%	190	62.7%
Symptoms of CTS usually begin slowly and overnight	18	5.9%	91	30.0%	194	64.0%
People with CTS are usually uncomfortable at night and in the morning	29	9.6%	85	28.1%	189	62.4%
Repeated physical activities like using computer and taping are major risk factors of CTS	19	6.3%	116	38.3%	168	55.4%
Avoid repetitive movement is a major preventive measure of CTS	36	11.9%	87	28.7%	180	59.4%
Surgical intervention is the main treatment of CTS	40	13.2%	69	22.8%	194	64.0%

Table 4 examines the relationship between knowledge and demographic factors of the patients. Significant differences were found in the knowledge of CTS based on gender (p = 0.001) and type of diabetes (p = 0.041). Females had a higher proportion of adequate knowledge compared to males, and participants with type 1 diabetes had a higher proportion of adequate knowledge compared to those with type 2 diabetes.

**Table 4:** The relation between knowledge and demographic factors of the patients

The relation between knowledge and demographic factors of the patients		Knowledge				
		Inadequate		Adequate		P-value
		N	%	N	%	
Age	18-28	35	57.4%	26	42.6%	0.192
	29-38	24	75.0%	8	25.0%	
	39-49	52	70.3%	22	29.7%	
	50 or older	97	71.3%	39	28.7%	
Gender	Male	93	79.5%	24	20.5%	0.001*
	Female	115	61.8%	71	38.2%	
Nationality	Saudi	193	68.0%	91	32.0%	0.317
	Non-Saudi	15	78.9%	4	21.1%	
Level of education	Non-learner	27	84.4%	5	15.6%	0.137
	High school	51	70.8%	21	29.2%	
	University student	20	52.6%	18	47.4%	
	Bachelor’s degree	78	68.4%	36	31.6%	
	Master’s degree	19	67.9%	9	32.1%	
	Doctor degree	13	68.4%	6	31.6%	
Marital status	Single	45	64.3%	25	35.7%	0.504
	Married	141	69.5%	62	30.5%	
	Divorced	11	84.6%	2	15.4%	
	Widow	11	64.7%	6	35.3%	
What type of diabetes do you have?	Type 1	75	62.0%	46	38.0%	0.041*
	Type 2	133	73.1%	49	26.9%	
Diabetic control	Uncontrolled	127	66.1%	65	33.9%	0.217
	Controlled	81	73.0%	30	27.0%	

In general, the mean BCTQ score among diabetic patients was 16.76 (SD=7.53). The severity of CTS was found to be significantly associated with various demographic factors and diabetic characteristics. The analysis revealed that age had a significant relationship with severity (p = 0.001*), with participants aged 39-49 and 50 or older showing higher mean BCTQ scores (17.95 and 17.89, respectively) compared to younger age groups. Gender also had a significant association (p = 0.027*), with females (mean BCTQ score of 17.52) exhibiting higher severity scores than males (mean BCTQ score of 15.56). Marital status showed a significant relationship with severity (p = 0.004*), with widowed individuals having the highest mean BCTQ score (22.35). Among diabetic characteristics, complications in the eye (p = 0.001*) and pain/numbness in the legs or feet (p = 0.000*) were significantly associated with higher severity scores. Participants who had heard of CTS (p = 0.000*) and had adequate knowledge about it (p = 0.000*) also showed higher severity scores. In terms of management, the use of medical interventions such as splints (mean BCTQ score of 19.29), injections around the wrist (mean BCTQ score of 25.90), and carpal tunnel release surgery (mean BCTQ score of 31.17) were associated with higher severity scores. In addition, there is no difference in BCTQ score between taking a specific type of insulin and not using it in all insulin types except for RYZODEG (mixed-insulin), in which using this type is associated significantly with a higher BCTQ score. Moreover, no significant difference was found between different types of insulin in the score of BCTQ (P=0.167) (Table 5).

**Table 5:** The relation between severity and management of CTS and demographic factors and diabetic characteristics BCTQ: Boston Carpal Tunnel Syndrome Questionnaire, CTS: Carpal tunnel syndrome

The relation between severity and management of CTS and demographic factors and diabetic characteristics		BCTQ score		P-value
		Mean	Standard Deviation	
Age	18-28	14.48	6.06	0.001*
	29-38	13.56	5.03	
	39-49	17.95	7.63	
	50 or older	17.89	8.17	
Gender	Male	15.56	6.20	0.027*
	Female	17.52	8.20	
Nationality	Saudi	16.78	7.49	0.856
	Non-Saudi	16.47	8.46	
Level of education	Non-learner	17.56	8.08	0.924
	High school	16.79	7.75	
	University student	15.92	6.60	
	Bachelor’s degree	16.75	8.05	
	Master’s degree	17.54	6.28	
	Doctor degree	15.84	6.64	
Marital status	Single	15.03	6.37	0.004*
	Married	16.91	7.52	
	Divorced	16.46	9.24	
	Widow	22.35	8.55	
What type of diabetes do you have?	Type 1	16.44	7.85	0.546
	Type 2	16.97	7.34	
What is the duration of the diabetes	Less than one year	14.19	6.16	0.052
	1-5 years	17.25	7.31	
	6-10 years	16.42	7.77	
	11-15 years	17.02	7.48	
	16-20 years	17.41	6.37	
	More than 20 years	18.76	9.10	
Diabetic control	Uncontrolled	17.17	7.65	0.216
	Controlled	16.05	7.32	
Have you ever heard of Carpel Tunnel Syndrome?	No	14.75	7.10	0.000*
	Yes	18.08	7.54	
Knowledge	Inadequate	15.27	6.42	0.000*
	Adequate	20.01	8.74	
What type of drug do you take for your diabetes?	Insulin injections	15.83	7.09	0.103
	Oral medications	17.30	7.76	
Were you diagnosed with any complications in your kidney function?	No	16.57	7.49	0.155
	Yes	18.77	7.90	
Were you diagnosed with any complications in your eye?	No	15.78	7.06	0.001*
	Yes	19.00	8.15	
Were you diagnosed with any complications like pain and numbness in your legs or feet?	No	15.71	7.33	0.000*
	Yes	19.01	7.52	
How often do you take vitamin supplementations like vitamin B?	Never	15.96	7.94	0.473
	Occasionally	17.25	7.20	
	Routinely	16.58	7.80	
Did you use any medical intervention to your wrist symptoms?	I did not use anything	15.94	6.81	0.000*
	Splint	19.29	8.14	
	Injections around the wrist	25.90	8.46	
	Carpel tunnel release surgery	31.17	11.09	
Type of insulin	Insulin aspart/insulin aspart protamine (Novomix^®^)	18.38	8.12	0.540
	Insulin aspart (Novorapid^®^)	16.16	7.58	0.472
	Insulin glulisine (Apidra^®^)	16.78	7.10	0.994
	Insulin lispro/insulin lispro protamine (Humalog 25/75^®^)	14.67	4.04	0.630
	Insulin glargine (Basalgar^®^)	18.60	6.83	0.396
	Insulin glargine (Lantus^®^)	15.84	6.88	0.429
	Insulin degludec (Tresiba^®^)	16.94	8.76	0.919
	Insulin degludec/insulin aspart (Ryzodeg^®^)	27.00	4.90	0.006*
	Insulin glargine U-300 conc (Toujeo^®^)	14.87	6.45	0.212
Insulin type	Short-acting	16.38	6.86	0.167
	Long-acting	14.79	6.87	
	Mixed acting	20.89	7.74	
	Short and long-acting	16.08	6.73	
	Mix and long-acting	10.00	.	
	Mix and short-acting	20.20	8.41	

## Discussion

The primary objective of the current research was to assess the extent of CTS in individuals with diabetes, while considering a range of demographic variables and diabetic attributes. The results of this study illuminate the correlations between these variables and the extent of CTS, offering significant contributions to the comprehension of the illness in relation to diabetes.

Age and gender, among other demographic variables, were discovered to be highly correlated with the severity of CTS. In comparison to younger age cohorts, older age groups, particularly those aged 39-49 and 50 or older, demonstrated higher average BCTQ scores. This finding is consistent with prior investigations that suggest CTS is more widespread and severe among the elderly [11-13] where the few previous population-based studies of CTS suggest a bimodal age distribution with a peak between ages 50 and 54 years, and a second peak between 75 and 84 years [11]. Potential contributors to the elevated severity scores include age-related alterations in tissues and an elevated probability of comorbidities among the elderly [14].

Gender disparities were also noted, whereby females exhibited severity scores that were higher in comparison to males. This result aligns with other research that has documented an increased incidence and severity of CTS in females [15-17]. One study showed that women had a five times higher risk for developing CTS than men [15]. Hormonal influences, anatomical distinctions, and disparities in lifestyle and professional conditions might all play a role in this gender gap [18]. Still, additional research is necessary in order to comprehensively comprehend the fundamental mechanisms.

A correlation was observed between marital status and the severity of CTS; people who were widowed demonstrated the highest average BCTQ scores. Diverse elements may exert an influence on this relationship. For example, individuals who are bereaved may encounter elevated levels of stress and reduced social support, both of which have the potential to adversely affect their general well-being and exacerbate the intensity of symptoms associated with CTS. Nevertheless, further investigation is required in order to clarify the precise mechanisms at play.

Diabetes is suggested to be associated with poorer outcomes after CTR [19-23]. This may seem reasonable, given the high incidence of sensorimotor neuropathy in diabetic patients. Higher severity scores of CTS were substantially associated with problems in the eye and pain/numbness in the legs or feet when diabetic features were examined. Consistent with prior research emphasizing the influence of diabetes complications on the onset and severity of CTS [8,24,25], these results support this notion. Indicative of more advanced stages of diabetes, the existence of comorbidities such as retinopathy or peripheral neuropathy may exacerbate symptoms of CTS by causing nerve compression in the carpal tunnel [8,26]. Effective management of these problems is of utmost importance in order to mitigate the impact of CTS in individuals with diabetes.

Additionally, participants' knowledge of CTS was assessed. The findings revealed a notable deficiency in knowledge, as the majority of respondents exhibited an insufficient understanding of CTS, encompassing its etiology, manifestations, repercussions, and therapeutic interventions. This emphasizes the necessity for focused educational campaigns aimed at enhancing diabetic patients' knowledge and comprehension of CTS. Acquiring more information can encourage the adoption of preventive measures, promote treatment-seeking behavior, and assist early identification.

Higher severity scores were connected with the utilization of medical procedures, including splints, injections around the wrist, and carpal tunnel release surgery, in terms of management options. This finding implies that those experiencing more severe symptoms of CTS are inclined to seek medical procedures in order to mitigate their pain and restore their ability to operate [27,28]. Nevertheless, the determination to administer a medical intervention ought to be grounded in a thorough evaluation that considers various aspects, including the intensity of symptoms, functional constraints, and the patient's reaction to conservative therapies.

An additional contribution to the body of knowledge is made by the study, which emphasizes the correlation between the severity of CTS and diabetic problems. Healthcare specialists consider the presence of ocular problems and pain/numbness in the legs or feet to be significant clinical indications of CTS. These symptoms underscore the need for thorough diabetes control in order to reduce the risk and severity of the condition.

The insufficient understanding of CTS among diabetes patients underscores the necessity for educational programs. By acknowledging this area of limited understanding, medical professionals can enable individuals to identify initial indications, pursue suitable treatment, and implement preventive actions. It is imperative to prioritize the distribution of precise information via diverse channels, such as educational resources, healthcare practitioners, and social media platforms.

This study offers an important contribution to our knowledge of the frequency and relationships between diabetes and CTS in the context of medicine. It clarifies the complex interplay between diabetic characteristics, knowledge gaps, and management approaches, as well as the varied ways in which CTS manifests in people with diabetes.

A limitation of this study is that it does not provide an exhaustive analysis of the relationships between CTS and diabetes. Important next stages for research include examining these relationships in more detail, exploring additional factors beyond the scope of this study that may impact CTS in diabetics, and evaluating the effectiveness of focused therapies. This study does not investigate variables like the influence of various diabetes management strategies on CTS, lifestyle choices, or genetic predispositions, which warrant further investigation to gain a comprehensive understanding of the topic.

## Conclusions

The consequences of this paper's findings hold significance for healthcare providers. They emphasize how addressing CTS in diabetic populations requires a thorough and interdisciplinary approach. Comprehending the complex connection between various variables influencing the severity of CTS in patients with diabetes requires an understanding of the diverse nature of these relationships in order to develop customized and appropriate treatment options. Finally, our research opens the door to a more sophisticated comprehension of the control and therapy of CTS in diabetic patients. In order to deliver better care, it highlights the necessity of an ongoing, in-depth investigation of these relationships. This could open up new directions for more successful, patient-centered interventions that could greatly enhance the quality of life for persons impacted by both disorders.
